# Supplementary material for: Growth Hormone Improves Adipose Tissue Browning and Muscle Wasting in Mice with Chronic Kidney Disease-Associated Cachexia
Source: Int J Mol Sci. 2022 Dec 4;23(23):15310. doi: 10.3390/ijms232315310 (PMC9740214; doi:10.3390/ijms232315310)
Supplement: Supplementary file 1 [file ijms-23-15310-s001.zip › ijms-1979348-supplementary.pdf]

Supplemental Table S1: Immunoassay information for blood and serum chemistry, muscle adenosine triphosphate content as well as muscle and adipose tissue protein analysis.

Blood & Serum chemistry	Assay information
BUN and creatinine	VetScan Comprehensive Diagnostic Profile, Abaxix, 500-0038 https://www2.zoetisus.com/content/_assets/docs/Diagnostics/package-inserts/VETSCAN-Comprehensive-Diagnostic-Profile-Package-Insert-LBL 02422.pdf

Muscle & Adipose tissue	Assay information
ATP Assay Kit (Colorimetric/Fluorometric)	Abcam, ab83355
HSL (phosphor Ser552) Cell based ELISA kit	antibodies.com, A102387
Human Growth Hormone ELISA Kit	Abcam, ab190811
JAK2 (phosphor-Tyr221) ELISA kits	Assaybiotech, CytoGlow™, CBP1593
JAK2 ELISA kits	Antibodies-online.com, ABIN5674893
Mouse Adipose Triglyceride Lipase, ELISA Kit	MyoBioSource.com, MBS750961
Mouse Hormone Sensitive Lipase, ELISA kit	MyoBioSource.com, MBS720763
Mouse STAT5, ELISA kits	MyoBioSource.com, MBS9354254
Phosphotyrosine STAT5, ELISA kits	RayBiotech, PEL-Stat5-Y-1
UCP1 ELISA kits	Aviva Systems Biology, OKCD02970
UCP3 ELISA kits	Aviva Systems Biology, OKEH05259

Supplemental Table S2: PCR primer information

Gene	Forward primer sequence	Reverse primer sequence
Atf3	GAGGATTTTGCTAACCTGACACC	TTGACGGTAACTGACTCCAGC
Atgl	GGATGGCGGCATTTCAGACA	CAAAGGGTTGGGTTGGTTCAG
Atp2a2	GAGAACGCTCACACAAAGACC	CAATTCGTTGGAGCCCCAT
Atrogin-1	CAGCTTCGTGAGCGACCTC	GGCAGTCGAGAAGTCCAGTC
Cidea	TGACATTCATGGGATTGCAGAC	GGCCAGTTGTGATGACTAAGAC
CD137	CGTGCAGAACTCCTGTGATAAC	GTCCACCTATGCTGGAGAAGG
Csrp3	GGGGGAGGTGCAAAATGTG	CAGGCCATGCAGTGGAAACA
Cyfip2	ATGACCACCCACGTCACTTTG	CCTGTCCTCGAAGTTCGTGTC
Cox2	AACCCAGGGGATCGAGTGT	CGCAGCTCAGTGTTTGGGAT
Fhl1	GACTGCCGCAAGCCCATAA	CCAAGGGGTGAAGGCACTT
Fos	TTGAGCGATCATCCCGGTC	GCGTGAGTCCATACTGGCAAG
Hsl	CCAGCCTGAGGGCTTACTG	CTCCATTGACTGTGACATCTCG
Gng2	ACCGCCAGCATAGCACAAG	AGTAGGCCATCAAGTCAGCAG
IGF-I	GTGGGGGCTCGTGTTTCTC	GATCACCGTGCAGTTTTCCA
IL1b	GCAACTGTTCCTGAACTCAACT	ATCTTTTGGGGTCCGTCAACT
Il6	TAGTCCTTCCTACCCCAATTTCC	TTGGTCCTTAGCCACTCCTTC
Itpr1	CGTTTTGAGTTTGAAGGCGTTT	CATCTTGCGCCAATTCCCG
Maff	AGGAGGAGGTCATCCGACTG	CTTCTCGCTCTCCAGAATGTG
Murf-1	GTGTGAGGTGCCTACTTGCTC	GCTCAGTCTTCTGTCCTTGGA
Myd88	TCATGTTCTCCATACCCTTGGT	AAACTGCGAGTGGGGTCAG
Myl2	ATCGACAAGAATGACCTAAGGGA	ATTTTTCACGTTCACTCGTCCT
Myod	CCACTCCGGGACATAGACTTG	AAAAGCGCAGGTCTGGTGAG
Myogenin	GAGACATCCCCCTATTTCTACCA	GCTCAGTCCGCTCATAGCC
Myostatin	AGTGGATCTAAATGAGGGCAGT	GTTTCCAGGCGCAGCTTAC
Pax7	TCTCCAAGATTCTGTGCCGAT	CGGGGTTCTCTCTCTTATACTCC
Pgc1α	TATGGAGTGACATAGAGTGTGCT	CAGGAGTTGATTCCAGACAGGTA
Pgf2a	CTGGACTCATCGCAAACACAA	AGGAAGCCTTTGACTTCTGTCTA
Prdm16	CCCCACATTCCGCTGTGAT	CTCGCAATCCTTGCACTCA
Socs-2	GCGCGTCTGGCGAAAGCCCT	GAAAGTTCCTTCTGGAGCCTCTT
Tbx-1	CTGTGGGACGAGTTCAATCAG	TTGTCATCTACGGGCACAAAG
Tlr2	GCAAACGCTGTTCTGCTCAG	AGGCGTCTCCCTCTATTGTATT
Tmem26	TTCCTGTTGCATTCCCTGGTC	GCCGGAGAAAGCCATTTGT
Tnfa	*CCCTCACACTCAGATCATCTTCT*	*GCTACGACGTGGGCTACAG*
Tnnc1	GCGGTAGAACAGTTGACAGAG	CCAGCTCCTTGGTGCTGAT
Tpm3	ACCACCATCGAGGCGGTAA	CCCTTTCCTCCGCATCATCA
Traf6	AAAGCGAGAGATTCTTTCCCTG	ACTGGGGACAATTCACTAGAGC
Gapdh (internal control)	AGGTCGGTGTGAACGGATTTG	TGTAGACCATGTAGTTGAGGTCA
